# Single-use *versus* conventional reusable flexible ureteroscopes – an evaluation of the functional parameters

**DOI:** 10.25122/jml-2022-0269

**Published:** 2023-01

**Authors:** Marius Bragaru, Razvan Multescu, Dragos Georgescu, Cătălin Bulai, Cosmin Ene, Razvan Popescu, Petrişor Geavlete, Bogdan Geavlete

**Affiliations:** 1 Urology Department, Sf. Ioan Emergency Clinical Hospital, Bucharest, Romania; 2 3^rd^ Department, University of Medicine and Pharmacy Carol Davila, Bucharest, Romania

**Keywords:** single-use, reusable, flexible ureteroscope, lithiasis

## Abstract

The purpose of single-use flexible ureteroscopes (su-fURS) was to overcome the limitations of conventional reusable ureteroscopes in terms of maneuverability and maintenance. We aimed to perform a systematic literature review on available su-fURS performance *versus* conventional reusable fURS focusing on clinical data. A systematic research using Pubmed was performed evaluating single-use fURS and reusable fURS in urinary tract stone disease, including prospective assessments and case series. This review aimed to provide an overview of single-use and disposable flexible ureteroscopes and to examine and compare their capabilities (deflection, irrigation, optical properties). We included 11 studies, where the single-use fURS were compared to the reusable fURS. The studies with single-use ureteroscopes included data on LithoVue (Boston Scientific), The Uscope UE3022 (Pusen, Zhuhai, China), NeoFlex-Flexible, (Neoscope Inc San Jose, CA), 23 YC-FR-A (Shaogang). For reusable ureteroscopes, data were included on three models, two digital (Karl Storz Flex-XC and Olympus URF-Vo) and one fiber optic (Wolf-Cobra). There were no significant differences in stone-free rate, procedure duration, or functional capabilities between single-use fURS and reusable fURS. The systematic literature review analyzed operative time, functional capabilities, stone-free rates, and postoperative complications of the ureteroscopes, and a special chapter about renal abnormalities to emphasize that they are a good choice having a high proportion of stone-free rates and few risks, particularly in treating difficult-to-access calculi. Single-use fURS demonstrate a comparable efficacy with reusable fURS in resolving renal lithiasis. Further studies on clinical efficacy are needed to determine whether single-use fURS will reliably replace its reusable counterpart.

## INTRODUCTION

Flexible ureteroscopes have come a long way since the first generation of analog devices, which were only capable of a certain amount of bending, up to the most recent digital technologies, which offer enhanced flexibility and dependability. The technical restrictions that previously existed in regard to visibility and access have been removed. Renal calculi can now be treated with flexible URS independent of the calyx groups involved. Moreover, ureteroscopy was only able to be performed using reusable ureteroscopes until quite recently [1].

Developing digital ureteroscopes that are reusable and have superior durability to that of fiberoptic models is an ongoing problem [2–4]. An increased number of surgeries might cause damages such as a loss of deflection and a decline in the performance of the scope for subsequent procedures [5]. Lastly, sterility is an important factor to consider when using reusable flexible ureteroscopes [5, 6]. Even when reusable ureteroscopes had been cleaned manually and sterilized by hydrogen peroxide gas, contamination (*e.g*., bacteria, adenosine triphosphate, hemoglobin, protein) appeared in some instances, as highlighted by Ofstead et al. in their research. As an alternative to reusable scopes, single-use flexible ureteroscopes (su-fURS) and semi-rigid ureteroscopes have been developed recently [6].

There are already over ten models of su-fURS on the market, each with its unique set of attributes and level of performance (Table 1). Some of these devices have characteristics that are similar to those of reusable flexible ureteroscopes, and others have even more advanced features.

**Table 1:** Some of the commercially available devices.

Single-use flexible ureteroscope	Manufacturer
**LithoVue**	Boston Scientific in Massachusetts
**Uscope**	Zhuhai Pusen Medical Technology Co. Ltd
**NeoFlex**	Neoscope Inc.
**Shaogang or YC-FR-A**	YouCare Tech
**PolyScope**	Polydiagnost GmbH
**Semi-Flex**	Maxiflex
**Flexor-Vue**	CookMedical

The purpose of this evaluation is to compare and contrast the functional capabilities of traditional reusable FURS with those of disposable single-use fURS.

## MATERIAL AND METHODS

**Figure 1:**
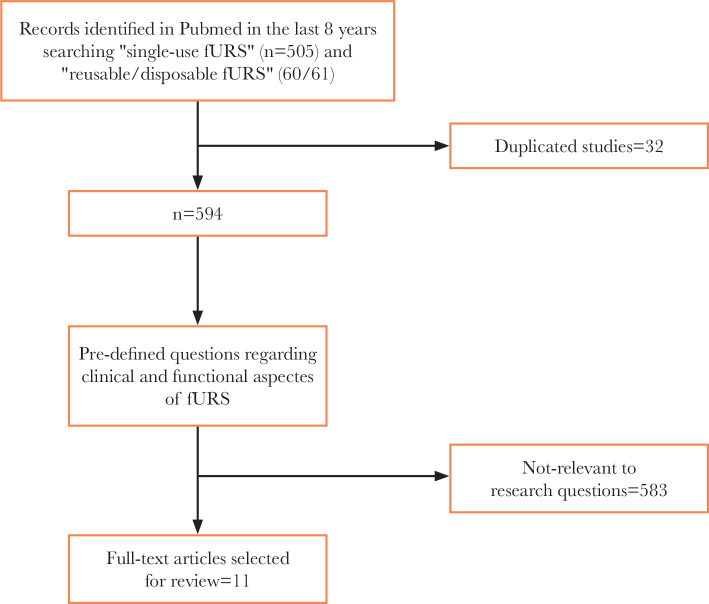
Selection criteria for the reviewed articles.

Systematic research using the Pubmed electronic database was performed for studies evaluating single-use and reusable fURS in the setting of urinary tract stone disease. Keywords included "ureteroscopy", "flexible ureteroscope", "reusable ureteroscope", "single-use/disposable flexible ureteroscopy", and "single (use)/disposable flexible ureteroscope". Eleven publications between 2013 and 2021 were examined, and if it was unclear from the abstract whether the paper would contain pertinent data, the whole paper was evaluated (Figure 1). The references cited in all full-text articles were also assessed for additional relevant or associated articles. Non-English articles were excluded from the analysis. With the consensus of the co-authors, the relevant studies were then selected and screened, and the data were extracted and analyzed. The systematic review tried to respond to predetermined questions:

1. Is there a difference between single-use and reusable flexible ureteroscopes in terms of operative time?
2. Are the rates of stone-free procedures and complications different for single-use and reusable flexible ureteroscopes?
3. What is the difference between the endoscope's functional capabilities?

The Preferred Reporting Items for Systematic Reviews and Meta-Analyses (PRISMA) flowchart was used to report the number of papers identified and included or excluded at each stage. We then performed a narrative review of relevant findings.

## RESULTS

### Operative time

Longer procedures are associated with greater risks of systemic inflammatory response syndrome (SIRS), fever, or sepsis, especially in patients with infectious stones [7].

For stones up to 20 mm in size, operational times for URS and retrograde intrarenal surgery (RIRS) ranged from 25 to 65 minutes [8].

More can be done in this time than in the allotted 90 minutes. Due to the elimination of blood and stone fragments, and the ability to increase perfusion flow rates with normal saline without exceeding essential renal pelvic pressure values, suction may help reduce surgical time. Suctioning more effectively to remove stone fragments is another way to reduce processing time [9].

In pigs, renal damage develops after 60 minutes of renal pelvic pressure >15 mmHg, and one cc of irrigation fluid is absorbed for every minute of RIRS [10]. Therefore, saving time is a major advantage of adopting suctioning technology.

Shiyong Qi and colleagues conducted a prospective study to evaluate the clinical results of the Olympus digital reusable fURS with the single-use digital fURS known as ZebraScope. The primary outcome was the percentage of patients who were stone-free one month after surgery (SFR). The trial involved 126 patients, with 63 participants assigned to each group. In the ZebraScope group, the one-month SFR was 77.78%, while in the URF-V group, it was 68.25% (two-sided 95% confidence interval [CI]: -5.95 to 25.01). According to the findings, there was not a significant difference in the amount of time spent operating between the two groups (p=0.687) [11]. In addition, the study by Kam et al. included 141 patients undergoing stone therapy, and it compared the reusable digital URF-V2 with the LithoVue and the PU3022A (Pusen, Olympus). They discovered no differences in the amount of time it took to operate [12]. According to Usawachintachit et al., the overall mean procedure duration for LithoVue was 10.4 minutes shorter than for fiberoptic URF-P6 (64.5 *vs*. 54.1 min; p 0.05). In stone removal procedures, this gap widened to 13 minutes and remained statistically significant (70.3 *vs*. 57.3 min; p 0.05), resultingin a shorter time spent in the operating room with LithoVueTM (104.3 minutes as opposed to 89.8 minutes; p<0.05) [13].

### Functional capabilities: mechanical, irrigation, optical properties, and access sheet importance

Our research included information about three different models of reusable ureteroscopes: two digital (Karl Storz Flex-XC and Olympus URF-Vo) and one fiber optic (Shaogang 23 YC-FR-A), and four models of single-use: LithoVue (Boston Scientific), The Uscope UE3022 (Pusen, Zhuhai, China), NeoFlex (Wolf-Cobra).

For intrarenal surgeries, the capacity of a flexible ureteroscope to deflect is essential since it is required for access and navigation of the renal pelvis, calyces, and diverticula [14]. Moreover, it is of the utmost importance while attempting treatment of lower pole calculi, as the lower pole of the kidney is more difficult to approach.

Scotland et al. compared the performance of one disposable digital ureterorenoscope called the Flex-XC to two single-use instruments called the LithoVue and the Uscope (Storz). According to the findings, the amount of deflection exhibited by the LithoVue and Uscope devices was superior to that of the reusable scope, both when the working channel was vacant and when it was being utilized. Image distortion was not significantly different between the LithoVue and the Uscope when compared to the Flex-XC reusable scope. While the LithoVue exhibited superiority in deflection and irrigation flow, the Uscope proved superiority in color representation and field of view. The Flex XC featured the highest image resolution of the two models. *In vivo*, each scope had an equal amount of success when it came to reaching the higher, middle, and lower poles. When accessing the collecting system, performing laser lithotripsy, and extracting stones using a basket, visibility was equivalent across all scopes [15].

Dragos and colleagues conducted an *in vitro* study in which they compared the performance of four disposable flexible ureteroscopes (LithoVue, Uscope, NeoFlex, and Shaogang) to that of four reusable flexible ureteroscopes. The majority of the single-use flexible ureteroscopes indicated general superiority in their deflection capabilities; the only areas in which the reusable ureteroscopes had stronger deflection capabilities were with the guidewires and the 365 m laser fibers. When compared within the category of single-use flexible ureteroscopes, the cumulative degrees of deflection (the sum of all deflection angles when the working channel was occupied) were largest with the NeoFlex, followed by the LithoVue, then the Uscope, and finally the Shaogang [16]. In addition, an ex-*vivo* investigation by Hennessey et al. compared the deflection capabilities of the LithoVue to those of two reusable ureteroscopes, namely the URF-V (Olympus, Tokyo, Japan) and the Flex-XC (Karl Storz&Co. KG, Tuttlingen, Germany), showed similar results [17]. Davis et al. studied *in vitro* characteristics of LithoVue, Polyscope, and SemiFlex and concluded that typical fiberoptic reusable flexible ureteroscopes have similar mechanical, optical, and irrigation qualities [18].

However, Tom et al. showed that the Shaogang ureteroscope had a higher flow rate than the digital Flex-Xc and the fiberoptic Cobra flexible ureteroscopes. This was discovered after comparing the Shaogang and NeoFlex single-use devices. The scientists conducted bench testing to assess the irrigation flow rates of the Flex-Xc reusable digital ureteroscope, the LithoVue, and the Uscope. They discovered that the LithoVue had superior flow rates both with an empty working channel and with a 273 m laser fiber [19].

Kam and colleagues concluded that the URF-V2 group had greater mobility scores than the PU3022A and better visibility scores than either of the single-use scopes. When compared to the PU3022A, the LithoVue scored higher than the PU3022A in terms of both visibility and maneuverability [12].

Ureteral access sheaths (UASs) are disposable medical devices used for stone retrieval by securing the scope's entry into the patient's upper urinary tract from the outside. In 1974, Hisao Takayasu and Yoshio Aso developed the first ureteroscope insertion aid (UAS) to "guide tube" the ureteroscope (URS) into the ureter. This first prototype was a polytetrafluoroethylene tube measuring 3 mm in diameter and 38 cm in length, into which a flexible URS was inserted [20].

Modern UAVs come in a wide range of sizes. The use of a UAS during ureteroscopic operations has various benefits, including the ability to make multiple passes of the instrument over the kidney and the reduced difficulty in manipulating the scope. To improve kidney vision at lower pressures, it can also modify the dynamics of irrigation flow.

A modified UAS was described by Zeng and colleagues. It featured an evacuation side port and was designed for use with a negative pressure vent that was connected to continuous negative suction. This was done to remove small fragments of stone during and immediately after ureteroscopic lithotripsy. The negative pressure values used by the authors ranged from 150 to 200 mm Hg. During the process of laser lithotripsy, a significant portion of the stone fragments would be suctioned through the side port. When stone fragments were too large to transit through this route, they could be removed from the sheath by retracting the scope until it reached the bifurcation of the connecting side port at the proximal end of the sheath. This made it possible for the stone to be removed through the side port. The initial stone-free rate (SFR) was 97% in 74 patients who were treated with the modified UAS for ureteral stones [21].

Zhu and his colleagues just recently described a separate unmanned aerial system (UAS) that is capable of suction. This sheath features two different tubes on its proximal end; one of them is for suction, while the other is for pressure venting. The inner obturator of the sheath is sized 12F, and the outer sheath is sized 14F. The authors conducted a matched-pair analysis on a total of 165 patients who were going through ureteroscopy to evaluate the results of using a suctioning ureteroscope with those of using a normal ureteroscope. The suction UAS cohort had a shorter mean operative duration (50 minutes *vs*. 57 minutes) and a greater immediate SFR on postoperative day 1 (83% *vs*. 72%). Both groups had comparable percentages of people who passed their stone after one month. The suction UAS cohort also had a reduced overall complication rate (12% compared to 25%) and fewer infectious postoperative problems [22].

### Stone-free rates and postoperative complications

Giorgio Bozzini and colleagues compared the efficacy and safety of reusable flexible ureteroscopes with digital technology (FLEX Xc, KARL STORZ SE & Co. KG, Tuttlingen, Germany) and disposable flexible ureteroscopes also endowed with digital technology (US31B-12, InnovexAnqing Medical Instrument CO. LTD, Shanghai, China) for patients undergoing Retrograde Intrarenal Surgery (RIRS). In total, there were 180 patients that participated in the research. Ninety patients received RIRS treatment using a reusable flexible ureteroscope (group A), and the remaining ninety patients received RIRS treatment using a disposable flexible ureteroscope (group B). The Stone Free Rates (SFRs) did not differ substantially between the two groups (86.6% for group A *vs*. 90.0% for group B, p=0.11), but the overall complication rate was significantly higher in group A at 8.8% than in group B at 3.3% (p<0.05). Specifically, group A showed a greater number of serious difficulties than the other groups (Clavien score IIIaV). The postoperative infection rate was significantly higher in group A at 16.6% than it was in group B at 3.3% (p<0.05) [23]. Also, Somani et al. compared reusable digital ureteroscopes to fiber optic flexible ureteroscopes and found comparable outcomes in terms of accessibility to the complete collecting system and stone-free rates (SFRs). The risk of complications was comparable across the two treatment approaches [24]. However, in a different study, Mager et al. prospectively compared 68 consecutive procedures using reusable flexible ureterorenoscopes (Flex-X2S/Flex-XC, Karl Storz) with 68 consecutive procedures using single-use digital flexible ureterorenoscopes. This comparison was made to determine which type of ureterorenoscope was more effective (LithoVue). Patients had the same number of stones in their bodies as well as similar demographic parameters. The authors found no statistically significant differences in SFR (82% *vs*. 85%; p=0.8) and complication rates (7 *vs*. 17%; p=0.06) with reusable and single-use scopes, respectively. However, as a paradox, one febrile urinary tract infection (UTI) case occurred in the single-use group and none in the reusable scope cohort [25].

A prospective study conducted by Shiyong Qi and colleagues came to the conclusion that digital single-use fURSs are an effective and safe alternative to reusable URF-V [11]. In the meanwhile, Usawachintachit et al. conducted prospective case-control research in which they compared Olympus fiberoptic URF-P6 to LithoVue. A total of 116 cases were completed using a single-use scope and 65 cases using a scope that could be reused. For LithoVue, the number of patients who had no fragments, negligible residual fragments (2 mm), and substantial fragments (>2 mm) was 60.0%, 12.5%, and 27.5%, respectively, while for URF-P6, those percentages were 44.7%, 13.2%, and 42.1% (p=0.36). In this particular research project, it was found that the single-use scope produced superior results (Table 2) [13].

**Table 2:** Single-use and reusable fURS, post-operative and technical parameters.

	Reusable fURS (Type)	Single-use fURS (Type)	Study
**Stone free rates**	86.6% (FlexXc)	90% (US31-B-12)	Giorgio Bozzini et al.
	68.25% (Olympus URF-V)	77.78% (ZebraScope)	Shiyong Qi. *et al*.
	44.7% (Olympus URF-P6)	60% (LithoVue)	Usawachintachit *et al*.
	82% (Flex-Xc| Flex-X2S)	85% (LithoVue)	Mager *et al*.
**Operative time**	64.3min	64.1min	Shiyong Qi. *et al*.
	64.5 min	54.1 min	Usawachintachit *et al*.
	76.2 min.	76.8 min.	Mager *et al*.
	72.3 min. (URF-V2)	86.1 min. (LithoVue)	Kam *et al*.
**Ureteroscope deflection with an empty working channel**	285° (Flex-Xc)	295° (LithoVue) 290° (Uscope UE3022)	Scotland *et al*.
	219° (Flex-Xc)	286° (LithoVue)	Hennessey *et al*.
		270° (LithoVue) 180° (PolyScope) 300°/265° down/up (SemiFlex)	Davis *et al*.
	263° (Flex-Xc)	276° (LithoVue) 226° (NeoFlex)	Tom *et al*.
**Visibility score**	4.2 (URF-V2)	4.8 (LithoVue)	Kam *et al*.
**Maneuvrability score**	4.7 (URF-V2)	4.9 (LithoVue)	Kam *et al*.

The stone-free rate is the primary effectiveness metric for surgical procedures used to control stone disease. The definition of stone-free rate varies from study to study, but it typically refers to there being no residual fragments larger than 2–4 millimeters after the procedure. Several studies demonstrated satisfactory percentages of stone-free rates while employing a variety of modified access sheets.

When treating ureteral stones in various segments of the ureter with their modified UAS, Zeng et al. reported an immediate SFR of 97.3 percent and a 1-month SFR of 100 percent [21].

Similar outcomes were observed during URS/RIRS procedures that included suctioning, with an immediate stone-free rate ranging from 87.5–95.7% and a delayed stone-free rate ranging from 92.5–95.6% [26, 27].

This percentage is in line with the findings of CROES and is lower than the 6.9 percent rate of postoperative fever (POF) showcased by Southern et al. [28]. Even for stones as large as 20–30 mm, this percentage was reduced to as low as 4.3% and as high as 13.1% when suctioning was utilized, and none of these patients developed sepsis [26, 27]. Postoperative fever following URS or RIRS accounted for 3.4–14.2% of all visits to the emergency room, and of these patients, over half needed hospitalization [28]. The terrible sequela of postoperative fever is sepsis, which has been linked to fatality rates as high as 41.1% [29]. Therefore, the use of suctioning can improve the safety of endourologic procedures even in more difficult situations by reducing the frequency of postoperative fever. Similar outcomes were observed during URS/RIRS procedures that included suctioning, with an immediate stone-free rate ranging from 87.5–95.7% and a delayed stone-free rate ranging from 92.5–95.6% [26, 27].

### Renal abnormalities

The selection of an appropriate therapeutic method for congenital abnormalities such as horseshoe kidney, ectopic pelvic kidney, and isolated rotation abnormality with accompanying lithiasis is still contentious. Due to the rarity of these instances, there was no consensus regarding the best course of treatment; however, multiple studies using ESWL, laparoscopy, PNL, and RIRS presented positive outcomes. Although it has been around for more than three decades, flexible ureteroscopy has recently emerged as an effective alternative surgical procedure for treating minor to medium-sized calculi, as well as some rare genetic conditions. This has made it possible for the procedure to become more effective. Recent studies have reported positive outcomes for patients who had retrograde intrarenal surgery [30–32].

The success rate when treating ectopic pelvic kidney was estimated to be 84.4% based on the findings of Bozkurt and colleagues. Their study examined the outcomes of flexible ureteroscopy procedures on 26 patients diagnosed with the same condition [33]. Ergin et al. compared the outcomes of retrograde intrarenal surgery and laparoscopy on patients with ectopic kidneys. The researchers found that the two types of interventions produced equivalent results [34]. According to other research, the success rate of flexible ureteroscopy in patients with pelvic kidney stones ranges anywhere from 75% to 84% [35, 36]. In a study by Geavlete et al., which described single-use flexible ureteroscopy for medium and large stones, the reached stone-free rate was up to 94.4% [37].

The horseshoe kidney is distinguished by an aberrant fusion of the kidney's lower poles and a heavily implanted ureter. Horseshoe kidneys more commonly affect males [38]. Weizer et al. reported some of the earliest attempts, in which they provided a stone-free rate of 75% for a limited series of malformations that comprised 4 HSK [35]. A few years later, Molimard et al. reported an 88.2% stone-free rate after 1.5 sessions per patient in their assessment of 17 instances of HSK treated using flexible ureteroscopy. The review included cases of patients who had previously been treated with traditional ureteroscopy [39]. Following an investigation of 25 surgical patients, Atis et al. reported a stone-free rate of 70% following one treatment session, with a mean stone burden of 17.8 4.5 mm [31]. According to the recent findings of Ding et al., an overall stone-free rate of 87.5% was attained, even for big calculi [40]. A recent study that compared the results of single-use *vs*. reusable ureteroscopes revealed a 92.25% stone-free rate for single-use when dealing with large stones with this kind of abnormality [41]. Similar results were also found in a second study by Geavlete B. et al. on renal malformations. Therefore, flexible ureteroscopy represents an effective therapeutic approach characterized by a remarkably high stone-free rate and few complications in Horseshoe kidneys and ectopic pelvic kidneys [37].

## CONCLUSIONS

In the treatment of renal calculi, the efficacy of single-use and reusable fURS is equivalent. Due to the reduced risk of ureteroscope damage, disposable scopes are especially effective for treating calculi that are difficult to access. To evaluate whether single-use fURS can reliably replace their reusable equivalent, additional clinical efficacy trials are required. Single-use flexible ureteroscopy is an excellent option for treating stones on congenital malformations such as an ectopic pelvic kidney horseshoe kidney due to its high stone-free rate and low risk.
